# Are Artificial Teeth Capable of Producing Salivation?

**Published:** 1871-08

**Authors:** P. A. O'Connell

**Affiliations:** Boston.


### ARTICLE VI.

### *Are Artificial Teeth Capable of Producing Salivation?*

By P. A. O'Connell, M.D., Boston.

My attention has been called to a case which points to the possibility of the occurrence of *salivation* and the *constitutional effects of mercury*, from the use of artificial teeth, and the importance of the circumstance has seemed to be sufficient to justify a mention of it; so that interferences may become either corrected or confirmed by the observations of others of the profession.

The patient, in the case referred to, was a lady, who had used the artificial teeth that are now accused of having produced trouble, between two and three years. Before using them, her general health was good. While using them her health became poor (*wasting away*), and proceeded gradually from bad to worse, resisting every mode of treatment. She exhibited no special cause of illness, until the occurrence of salivation and sore mouth drew attention to the teeth. Then it was found that the plate upon which the teeth were mounted, which was a suction plate of the red rubber kind, presented a corroded appearance on the surface which came in contact with the roof of the mouth. And the circumstance that this kind of rubber plate is made up to a great extent of the sulphuret of mercury, suggested the possibility of the general ill health resulting from this cause.

The teeth were removed, of course. The mouth became well speedily; and without any further treatment the lady's general health began to improve immediately in a very remarkable manner.

Upon mentioning this case to some medical gentleman, it recalled to the mind of one of them another instance of salivation, resulting apparently, from the same cause. Here too, the disuse of the red rubber plate allowed the mouth to become well; and a set of teeth mounted on dark rubber was used afterwards without any inconvenience resulting.

The red rubber which is used in making the plates upon which artificial teeth are mounted, receives its color from the sulphuret of mercury, which is mixed with it very intimately, and constitutes generally about one-third of the mass. This preparation of mercury is very insoluble, resisting, in the chemist's laboratory, the strongest acids; and it is difficult to understand what combinations can have taken place in the mouth to render it liable to absorption.

It is rendered soluble by mixture with the sulphide of potassium, but one would suppose that it would be protected sufficiently by the rubber with which it is thoroughly mixed and baked.

Are artificial teeth under any circumstances, capable of producing salivation?—*Boston Med. and Surg. Journal.*

